# Toward the Development of Combined Artificial Sensing Systems for Food Quality Evaluation: A Review on the Application of Data Fusion of Electronic Noses, Electronic Tongues and Electronic Eyes

**DOI:** 10.3390/s22020577

**Published:** 2022-01-12

**Authors:** Rosalba Calvini, Laura Pigani

**Affiliations:** 1 Department of Life Sciences, University of Modena and Reggio Emilia, Pad. Besta Via Amendola 2, 42122 Reggio Emilia, Italy; rosalba.calvini@unimore.it; 2 Department of Chemical and Geological Sciences, University of Modena and Reggio Emilia, Via G. Campi 103, 41125 Modena, Italy

**Keywords:** data fusion, artificial sensors, electronic tongue, electronic nose, electronic eye, food quality

## Abstract

Devices known as electronic noses (ENs), electronic tongues (ETs), and electronic eyes (EEs) have been developed in recent years in the in situ study of real matrices with little or no manipulation of the sample at all. The final goal could be the evaluation of overall quality parameters such as sensory features, indicated by the “smell”, “taste”, and “color” of the sample under investigation or in the quantitative detection of analytes. The output of these sensing systems can be analyzed using multivariate data analysis strategies to relate specific patterns in the signals with the required information. In addition, using suitable data-fusion techniques, the combination of data collected from ETs, ENs, and EEs can provide more accurate information about the sample than any of the individual sensing devices. This review’s purpose is to collect recent advances in the development of combined ET, EN, and EE systems for assessing food quality, paying particular attention to the different data-fusion strategies applied.

## 1. Introduction

The growing interest on the part of producers and consumers in the qualitative attributes of food has made it necessary to develop increasingly efficient analytical methods for monitoring the quality of the final product [1].

The overall sensory evaluation of food can be analytically studied using sensory methods and techniques. Objective methods for quality assessment include instrumental analysis, but to be of practical use for the food industry, instrumental methods must be cost-effective and provide rapid and reproducible results. In this context, the use of sensing systems, such as electronic noses (EN) [2,3], electronic tongues (ET) [4,5], and electronic eyes (EE) [6,7], can be an advantageous solution for the in situ study of real matrices, allowing to reduce sample manipulation. The final goal may consist of the estimation of overall quality parameters, also related to sensory characteristics, given by “smell”, “taste”, and “color” of the analyzed sample or in the quantitative determination of analytes (Table 1). Concerning ENs and ETs, gravimetric, optical, and electrochemical sensors can be used for analyzing volatile compounds or liquid samples [8,9,10,11]; however, electrochemical sensors are the most common sensing systems. Moving to EEs, traditional methods used to objectively determine food color properties involve the use of colorimeters and spectrophotometers, while in recent years, the use of computer vision systems based on red–green–blue (RGB) cameras has rapidly emerged [12].

When using ENs, ETs, and EEs, the approach followed in the determination of the parameters of interest is based on “blind analysis” techniques: the measured signals are analyzed with chemometric methods, which do not require any assumptions about the species to which a given pattern is ascribed [13,14,15]. Indeed, using this kind of approach, it is not necessary to assign the single details of the response in order to obtain coherent and useful results.

There are several reviews on the subject of ENs and ETs, and a discrete number on the more recent use of EEs, while the use of combined systems is less frequently reported. In principle, the combination of data acquired by ETs, ENs, and EEs, through proper data-fusion techniques, can furnish more accurate information about a sample than any one of the individual sensing devices [16,17] in the same way that the human brain combines the information resulting from multiple senses in order to gain a more accurate knowledge about a given object. Data-fusion techniques are categorized in different ways, according to the field of application in which they are involved, and they are nowadays frequently used in chemometrics when the combination of different analytical techniques is employed [18,19,20,21]. In the field of artificial sensors, they have been frequently adopted [22,23,24,25,26,27] in the case of combined ET and EN sensing systems, whereas the use of combined systems including an EE sensing system is less common [28,29,30].

Therefore, this review will concentrate on applications of combined EN, ET, and EE systems exclusively in the food sectors, with a particular focus on the application of the different data-fusion strategies.

## 2. Artificial Sensors

### 2.1. Electronic Nose

An accepted definition of an EN is “an instrument which comprises an array of electronic chemical sensors with partial specificity and an appropriate pattern recognition system, capable of recognizing simple or complex odor” [31].

Differently to other analytical instrumentation, ENs allow the identification of simple or complex volatile aroma mixtures as a whole, without the necessity to identify the individual chemical species within the sample mixture.

The EN technology is inspired by the sense of smell. The human olfactory system contains thousands of receptors that bind odor molecules; only in a few cases are there olfactory receptors which are specific for individual chemical molecules. Any given molecule may stimulate a combination of receptors, and some of the receptors can bind more than one odor molecule, creating a huge number of combinations that send unique signal patterns to the human brain. Most odorants are identified through a synthesis of the global chemical information from nonspecific interactions. The brain then interprets these signals and makes a judgment and/or classification to identify the consumed substance based on previous experiences or neural network pattern recognition.

To mimic the nonspecific recognition, the electronic nose often consists of nonselective sensors that interact with volatile molecules; upon interaction, a signal is produced which constitutes a sort of fingerprint of the odor. The signal is then used by an appropriate pattern recognition system to identify the odor through comparison with a reference library of previously obtained measurements of known samples. In this case, the nonselectivity of the sensors results in many possibilities for unique signal combinations, patterns, or fingerprints.

An EN is composed of three main components: the sampling system, the sensing system, and signal processing system. The sensor array can be considered the most important component of the EN. The sensors employed should have the highest sensitivity to the target group of chemical compounds. Moreover, the EN sensors should have relatively low selectivity to be sensitive to a wide number of different chemical compounds, coupled with rapid response and recovery times. As ENs are often used in open environments, a low sensitivity to temperature and humidity is also required.

The basis of gas sensor operation involves interactions between gaseous molecules and the sensor coating material, which determines selectivity; then, different transduction principles can be exploited. The most common sensors utilize transduction principles based on electrical measurements, including changes in current, voltage, and resistance; others involve mass changes, temperature or heat generation, and others are based on optical properties.

The most widely used class of gas sensors in ENs technology are metal oxide sensors (MOSs), and they form the bases of the more successful commercial products. The detection process involves the change in oxide conductivity in the presence of an oxidizing or reducing gas due to the reduction/oxidation reactions occurring at the oxide surface. Recent advances in artificial olfaction devices based on this kind of sensors are reported in the paper by Jeong et al. [32]. Conducting polymers are the other most used sensors in ENs. The swelling of polymers due to adsorbed chemical species can change the electrical properties of conducting polymers. Gravimetric sensors can also be used: the operation principle relies on the variation of the fundamental oscillation frequency of a thin quartz crystal as a result of the adsorption of gas analyte on its surface, which changes the oscillating mass. The surface of these sensors can be modified so that they can vary their selectivity [33].

Accurate description of the main sensor types used in ENs can be found in recent reviews [34,35,36]. Review reporting advances in EN sensors in food applications are also present in the literature [37,38,39,40].

### 2.2. Electronic Tongue

ETs can be defined as “multisensory systems for liquid analysis based on chemical sensor arrays and pattern recognition” [41]. Similar to ENs, the basic principle is to combine signals from nonspecific sensors with pattern recognition system, but at difference with ENs, the analyzed samples are liquid.

The original aim of ETs was to mimic the functions of human taste receptors: the recognition of the taste itself, rather than the discrimination of each chemical substance, is the final goal; for this reason, the device has also been defined as a “taste sensor” or an “artificial tongue” [42]. In this case, the array of sensors is used to classify a wide range of matrices into groups that reflect combinations of the five basic gustatory sensations, i.e., sweetness, sourness, saltiness, bitterness, and “umami”. This is not a trivial task, as taste can be elicited by compounds with very different structures and chemical properties. Moreover, the ETs may respond to the aroma compounds, which are dissolved in the liquid.

The same approach, i.e., the use of an array of sensors complemented by an appropriate pattern recognition system, has been then employed to distinguish between different liquid mixtures performing tasks such as recognition, classification, process monitoring, qualitative analysis, and even quantitative analysis. These more general systems are usually called “electronic tongues”.

The most important part of an ET is the sensor array: it comprises a set of chemical sensors with only partial selectivity to different compounds, the variety of selectivity providing a great deal of complementary information, i.e., high overall information content. Optimizing the choice of the sensor ensemble leads to the best information and can minimize the presence of noise and useless information. A number of chemical and biochemical (enzymatic) sensors exploiting various sensing techniques have been employed in designing ETs: electrochemical (potentiometric, voltammetric, amperometric, impedimetric, conductimetric), which are the most used, optical, and gravimetric [43]. In particular, electrochemical sensor arrays have proven to be highly efficient in discriminating complex liquid mixtures [44]. It is possible to obtain sensors with different sensitivity by chemical modification, reaching significant cross-selectivity.

Potentiometric sensors were used in the first studies on the application of sensor arrays for multicomponent analysis of liquids [45,46,47] and they still remain the most widely used type in the e-tongue systems, especially ion-selective electrodes (ISEs). The main disadvantage of potentiometric sensors is that they respond only to the charged species in solutions, which limits the number of potential analytes. The temperature variations and the adsorption of components present in the solution that can affect the membrane potential can be minimized by controlling the temperature and washing the electrodes. On the other hand, the advantages of ISEs are their well-known operation principle, low cost, simple setup, easy fabrication, and the possibility of obtaining sensors selective to many various species.

Voltammetric sensors are also widely used in ET systems. These devices are advantageous for multicomponent measurements because of their high selectivity, high signal-to-noise ratio, low detection limits, and various modes of measurement. Furthermore, the surface of the electrodes can be modified with various chemosensitive materials, obtaining sensors of various sensitivity and selectivity towards a variety of species. However, their applicability is limited to redox-active substances.

Principal applications of ETs in food analyses are reported in the literature [48,49,50,51].

### 2.3. Electronic Eye

**Figure 1:**
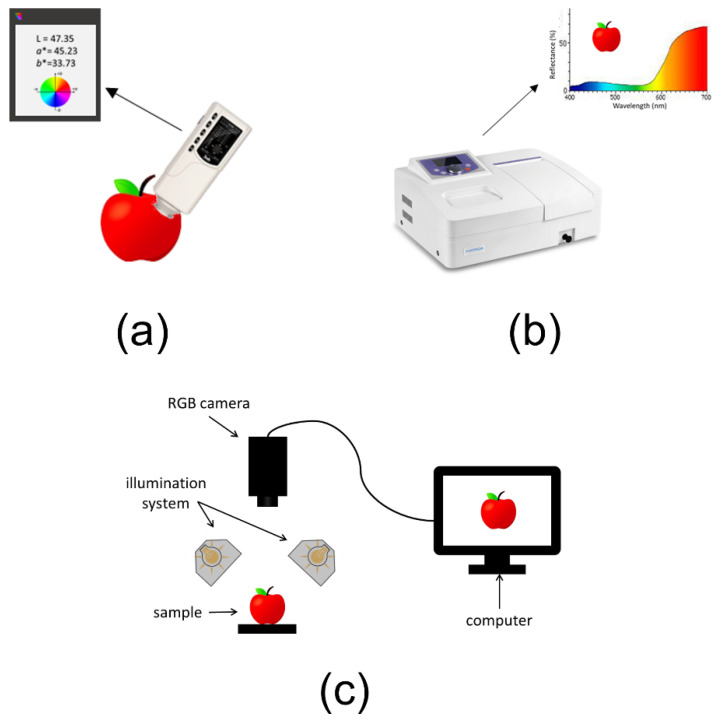
Representation of the different devices used as EE sensors: colorimeter (**a**), spectrophotometer (**b**), and computer vision system (**c**).

The EE is an analytical device designed to mimic human visual perception and to acquire color- and aspect-related information from a sample, allowing to gain an objective evaluation of the color properties of an object. Usually, EE sensors are based on colorimetry, spectrophotometry, or computer vision (Figure 1).

When using an EE to acquire color information from a sample, two complementary aspects should be considered: the choice of the proper device to measure color, and the choice of the proper manner to describe color.

Color is described using a color space, which is a mathematical representation able to associate color coordinates with each perceived color. There are three different types of color spaces: hardware-oriented color spaces, human-oriented color spaces, and instrumental-oriented color spaces [7]. Hardware-oriented spaces are used for hardware processing, such as, e.g., image acquisition or image display on digital screens. The most popular hardware-oriented space is the RGB space, which is defined by the coordinates on the red (R), green (G), and blue (B) axes. Human-oriented spaces better reflect color perception by the human eye, as we are more prone to describe color characteristics of an object using the concepts of tint, shade, or tone rather than the amount of red, green, and blue. An example of human-oriented space is the hue (H) –saturation (S) –intensity (I) space, where the concept of hue is related to the “purity” of a defined color, saturation is related to the perception of colorfulness of an area in relation to its brightness, and intensity describes the amount of light present in a color. Instrumental spaces are used by classical instruments measuring color, such as colorimeters, and they have been standardized by the Commission Internationale d’Eclairage (CIE) in order to have device-independent color coordinates. In color measurement of food products, the most used color space is the L*a*b* color space (also known as CIELAB).

Considering the analytical devices able to measure color, colorimeters and spectrophotometers are the most widespread tools used in the food industry. Colorimeters are composed of a light source, monochromatic filters, and a detector to spectrally emulate the sensitivity of the human eye, and the results are usually expressed in terms of CIELAB coordinates. On the other hand, spectrophotometers are able to register the whole spectrum of a sample in the visible range and then, using mathematical transformations, it is possible to calculate color coordinates of the sample, including L*a*b* values.

These two instruments allow to measure color properties of the analyzed samples considering only a limited surface area, resulting as less effective when it is necessary to evaluate color variability on larger sample areas or to analyze inhomogeneous samples. To overcome these limitations, computer vision systems are gaining an increasing interest in the objective evaluation of visual aspects of food products. Computer vision systems are composed of an illumination system, a digital RGB camera, a sample holder, and hardware and software for image acquisition and processing [52]. RGB cameras are based on charge-coupled device (CCD) sensors or on complementary metal-oxide Ssemiconductor (CMOS) sensors; essentially, both are arrays of minute photosensitive elements able to convert the intensity of incident light into an electric signal. In both cases, the sensor is covered by a mosaic of red (at λ ≈ 630 nm), green (at λ ≈ 545 nm), and blue (at λ ≈ 435 nm) filters, corresponding to the wavebands to which the human eye is sensitive. In the resulting RGB images, each pixel contains three integer values ranging from 0 to 255, corresponding to the red, green, and blue channels. Therefore, RGB images are three-dimensional data arrays with a size {r, c, 3}, where r is the number of pixel rows, c is the number of pixel columns, and 3 corresponds to the R, G, and B channels.

RGB images are complex data arrays, and it is fundamental to identify proper strategies to extract the useful information from such data [53]. Furthermore, when used in combination with signals derived from other sensors, it is necessary to compress the information contained in each three-dimensional RGB image into a one-dimensional signal, performing the so-called data-dimensionality reduction [20]. As a very straightforward method, average values of the three R, G, and B channels, or average values of color descriptors, such as, e.g., H, S, and I values, can be calculated from all the pixels of the image [54]. It is also possible to convert RGB images in L* a* b* values using proper calibration functions to relate the sensor response of the camera with CIE color-matching functions [55,56].

In order to preserve information related to spatial variability contained in the images, a possible strategy for image-data reduction consists of the calculation of histograms derived from one or more color parameters. Then, the histogram data are used as a color fingerprint signal of each image of the dataset [57,58].

## 3. Data Fusion

**Figure 2:**
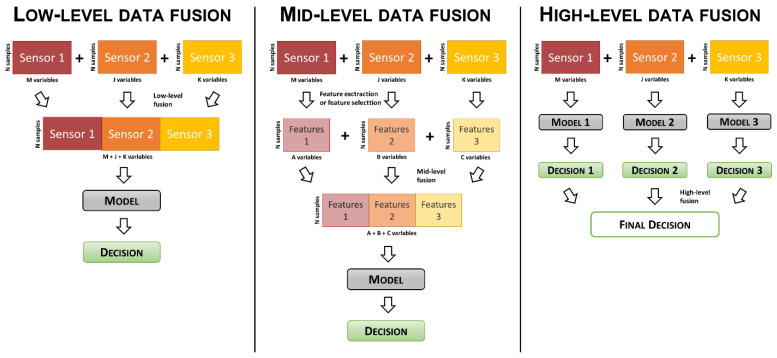
Schematic representation of the different data-fusion strategies.

The sensor technologies mentioned in Section 2 are able to provide information about different, and usually complementary, aspects of the considered set of samples. Data deriving from these different information sources can be jointly analyzed in order to gain a more comprehensive knowledge about the problem at hand. Fusion of data collected on the same sample set using different analytical sensors can be carried out at three levels: low-level, mid-level, and high level (Figure 2).

### 3.1. Low-Level Data Fusion

Low-level data fusion (or concatenated data fusion) represents the easiest and most straightforward way to jointly analyze multiple data blocks coming from different analytical sensors. In low-level data fusion, the variables obtained from the different sensors are simply merged row-wise, and the resulting data matrix has as many rows as the number of analyzed samples and as many columns as the sum of the number of variables of each data block. Then, this merged data matrix can be used to build multivariate calibration or classification models.

A key aspect of low-level data fusion is preprocessing, which is generally performed in two subsequent steps: firstly, each data block is separately preprocessed, and then scaling procedures are necessary for proper concatenation of the different data blocks.

In the first step, each block of signals is separately preprocessed in order to reduce the effect of noise or uninformative systematic variations. According to the nature of the acquired signals and of the analyzed samples, different preprocessing methods can be applied, for example Savitzky–Golay smoothing can be used to correct noisy signals [59], derivatives allow to correct baseline offsets (vertical shifts) and drifts (slope variations), as well as to enhance resolution of overlapped peaks [60], while global intensity effects can be corrected using standard normal variate or multiplicative scatter correction [61,62].

Considering the second step, adequate scaling or weighting procedures are necessary to account for the different dimensionality of the data blocks. As a matter of fact, when the data blocks have a very different number of variables, the outcomes of the analysis are strongly influenced by the largest block if proper scaling procedures are not carried out. To solve this issue, the most common preprocessing method applied to low-level merged data is block scaling, which consists of scaling each data block by its global standard deviation. In this manner, while preserving the relative weights of the variables within each block, the subsequent calculation of multivariate models is influenced by the different data blocks with equal weight [18].

Low-level data fusion has the main advantage of allowing a direct interpretation of the results in terms of the contribution of the original variables, and the correlation between variables belonging to different blocks can also be easily investigated. On the other hand, the noise content of the different data blocks is added.

### 3.2. Mid-Level DATA Fusion

In mid-level data fusion (or feature level data fusion), the original signals are separately analyzed to extract or select relevant features, and these features are then concatenated to obtain the fused dataset.

Two approaches can be adopted to obtain the features of interest from original signals: variable selection or feature extraction.

Variable selection approach consists of selecting the most relevant variables from each data block using variable selection algorithms, which automatically identify useful variables and discard the uninformative ones based on model predictions. Variable selection methods can be grouped into three main categories: filter methods, wrapper methods, and embedded methods [63]. In filter methods, a multivariate model is fitted on the input data and the important variables are selected by introducing a threshold on a measure of relevancy of the model itself, such as, e.g., the regression coefficients or the variable importance in projection (VIP) values [64,65]. Wrapper methods extract subsets of the original variables and evaluate the relevance of each subset by fitting a model to the extracted variables. The methods iterate between model fitting and variable selection in order to optimize model performances. Genetic algorithms (GA) [66] and interval-based methods [67] are examples of wrapper methods for variable selection. Finally, in embedded methods, variable selection is an integrated part of the modified classification or regression algorithm. For example, in sparse methods, variable selection is performed by introducing a penalization term on the objective function of the considered algorithm [68].

On the other hand, mid-level data fusion based on the feature extraction approach consists of retaining the relevant information contained in each set of signals using few latent variables accounting for underlying variable correlations and discarding noise. In this case, the features extracted from each data block consist of score vectors calculated from unsupervised or supervised methods based on latent variables, such as, e.g., principal component analysis (PCA), partial least squares (PLS) regression, or partial least squares-discriminant analysis (PLS-DA).

The extracted or selected features of each block are then concatenated, and the resulting dataset is analyzed with multivariate statistical methods to provide the final classification or calibration output.

When using mid-level data fusion based on subsets of the original variables, the interpretation of the results can be easily conducted as for low-level data fusion. However, the application of feature extraction methods is usually recommended in order to drastically reduce the number of variables considered in the merging procedure, and noise is discarded [18].

### 3.3. High-Level Data Fusion

Conversely to low-level and mid-level strategies, in high-level data fusion, the information related to the different sensors is combined at the decision level, and this kind of approach is mainly used for classification purposes.

More in detail, separate models are independently calculated for each block of signals, and the predictions obtained from the individual models are joined together to give the final decision. Different strategies can be employed to combine the predictions resulting from the single models to obtain the final output, from simple majority voting [69] to more complex methods based on Bayesian statistics [70] or evidence theory [71].

The main challenge in high-level data fusion consists of the identification of the optimal classification model for each block so that the combination of the outputs performs better than individual models [1].

## 4. Applications

### 4.1. EN + ET

EN and ET are used to obtain the complete flavor profile of foodstuff, which is the combined effect of the olfactory and gustatory aspects. In fact, the EN can evaluate volatile compounds or the aroma of a liquid in the headspace (i.e., evaluating the strength of the aroma concentration), while the ET can discriminate the concentration in a complex solution of the active compounds, which can affect the taste properties. Then, the combination of the two devices can successfully provide a complete characterization of the flavor of a food sample and, exploiting data-fusion techniques, important targets can be reached, such as the classification of similar products, the recognition of adulteration processes, and the definition of the degree of freshness (Table 2).

Zhang et al. used a self-developed ET and EN for evaluating the marked ages of rice wines [72]. The ET consisted of three types of modified electrodes with conducting polymer, while the EN constituted 12 MOS sensors and it was connected to a smartphone. Six types of feature datasets (ET dataset, EN dataset, direct-fusion dataset, weighted-fusion dataset, optimized direct-fusion dataset, and optimized weighted-fusion dataset) were used for identifying rice wines with different wine ages; the weighted-fusion data gave best results.

Good classification results were achieved also by Dong et al. in the analysis of seven Chinese Robusta coffee cultivars with different roasting degrees [73]. A fusion of both commercial EN and ET data was demonstrated to be an effective and powerful method for the rapid and nondestructive determination of coffee beans using the low-level data-fusion strategy. In particular, the combined data from the EN and ET performed much better than either approach alone in measuring the quality parameters of Chinese Robusta coffee beans when PLSR regression was used. Banerjee et al. applied ET and EN mid-level data fusion to classify black tea samples on the basis of their flavor characteristics, first exploiting a Bayesian approach [22] and then wavelet packet decomposition [74]. ET consisted of five electrodes made of five different noble metals and EN consisted of five MOS sensors composed of commercially available gas sensors. From the results, it was found that combined sensor response could classify black tea samples more accurately (99.75% classification rate) than individual utilization of EN or ET. Zakaria et al. employed low-level data-fusion techniques to discriminate different commercial *Orthosiphon stamineus* tea product samples, demonstrating that both PCA and linear discriminant analysis (LDA) results were improved by data fusion [75]. Combining a voltammetric ET with a low-cost EN, virgin olive oils were classified according to their geographical origins, as reported by Haddi et al. [23]. A perfect recognition was achieved by PCA, cluster analysis (CA), and support vector machine (SVM) when an improved low level was developed (low level of abstraction coupled with ANOVA variable selection).

In order to distinguish the organoleptic characteristics of minced mutton adulterated with different proportions of pork, low-level and mid-level data-fusion methods were applied to signals obtained from commercial EN and ET [76]. Hong at al. followed six approaches (two EN measurements, one ET measurement, and three fusion approaches using both instruments) for recognition and quantitative analysis of four unadulterated tomato juices and three adulterated tomato juices with different adulteration levels [77]. The first fusion approach was based on simple concatenation of original EN and ET sensors, the second fusion approach was based on stepwise selection, and the last one was based on an ANOVA-selected variable, which presented the best authentication performance. Men and al. used fusion technology based on the EN and ET to detect the blending ratio of the old frying oil and the new edible oil [78]. The characteristic vectors of both systems separately extracted from the two data blocks were used to form high-dimensional data as the new characteristics of the fusion system.

A low-level data-fusion model to combine EN and ET was developed by Dai et al. to detect submerged fermentation of *Tremella aurantialba* (*T. aurantialba*) [79]. The data were converted into 2D or 3D coordinates with irrelevant projection vectors, which retained the most important information from the original data. SVR models were used to establish the relationship between the data fusion of ET and EN and chemical indicators for the submerged fermentation values for quantitative prediction, showing high correlation degree.

### 4.2. EN + EE

As shown in Table 3, EN and EE are mainly applied for the determination of quality and safety attributes of food products such as meat, vegetables, or fruits. The combined use of these two devices allows to monitor, at the same time, the evolution of volatile compounds and color modifications caused by chemical changes of the analyzed matrix. Odor and color are strongly linked in the evaluation of food freshness, in particular when considering perishable food products. Indeed, chemical reactions occurring during food spoilage determine both the formation of peculiar volatile compounds and color modifications.

Korel at al. demonstrated the possibility of coupling EN and EE to determine the spoilage level of tilapia fillets treated with different percentages of sodium lactate and stored at different temperatures [81]. Electronic nose readings and color features of the analyzed samples were fused at the low level and used to develop a classification model able to assign the tilapia fillets to the correct spoilage class, obtaining higher classification rates compared to single sensors.

Considering meat products, total volatile basic nitrogen (TVB-N) content is a reference index to assess the freshness of pork meat. Huang et al. coupled EN and EE with near-infrared (NIR) spectroscopy to measure TVB-N content of pork meat samples [80]. The data analysis workflow followed by the authors can be summarized in three main steps: (i) extraction of the characteristic variables from each sensor response; (ii) application of PCA to each data block containing the characteristic variables of the three sensors to reduce the data dimensionality; (iii) mid-level data fusion of the score vectors of each PCA model and application of artificial neural networks (ANN) to the fused dataset to predict TVB-N content.

Liu et al. coupled the information resulting from a commercial portable EN and a Vis-NIR hyperspectral imaging (HSI) system (400–1000 nm spectral range), acting as an EE, in order to predict fungal contamination in strawberries [84]. Indeed, EE resulted as an effective method to monitor changes in exterior appearance and chemical composition (mainly total soluble solids and titratable acidity) in infected strawberries during storage. On the other hand, EN allowed capture of characteristic odor/aroma modifications of strawberries ascribable to fungal metabolism. Given these considerations, the two data blocks were merged at the mid-level by a preliminary compression of the two sensor datasets using PCA. Then, a calibration model was developed using mid-level fused data in order to predict fungal contamination, obtaining satisfactory results.

Color and aroma also characterize the quality and sensory attributes of food matrices, and combined EN and EE systems were successfully used to quantify quality parameters in different food matrices, such as quality levels of green tea [82], hardness and ripeness of tomatoes [83], and intramuscular fat and peroxide values of pork meat [85].

Xu et al. [82] developed a rapid classification method based on a commercial e-nose and computer vison to discriminate tea samples according to quality grading. To perform mid-level data fusion, relevant features were separately extracted from EN and EE data blocks using PCA. The resulting score matrices were merged together, and SVM algorithm was used for classification. In addition, high-level data fusion was also tested. In this case, two separate classification models were calculated for EN and EE data blocks using SVM, and the predictions obtained from the independent model were combined to obtain the final classification output.

### 4.3. ET + EE

Combination of EE and ET systems has been mostly applied to the analysis of liquid or semiliquid food samples characterized by chromatic characteristics, such as wines and honey. On the one hand, the ET can give rapid information about the chemical composition of the complex sample, including information about pH value or sugars, ethanol, and amino acids content. On the other hand, the EE can give useful information about color attributes, which can affect the visual sensory characteristics but also provide important qualitative parameters employed by farmers to estimate the time of the harvest or by the producers to control the quality of the final product or to modify the production process.

Table 4 lists the main applications of combined EN and EE analytical systems.

Gutierrez et al. deeply studied the potentialities of a multisensor consisting of a colorimetric optofluidic system and an array of electrochemical sensors for the characterization of red and white wines [86,87,88]. In their most recent work, the ET system comprised potentiometric, amperometric, and conductimetric sensors while the EE device consisted of a lab-on-a-chip spectrophotometer. Through a mid-level data-fusion method with feature selection by PCA and soft independent modeling class analogy (SIMCA), good classification of the grape varieties and identification of the mixtures were achieved. Moreover, using the PLS regression, the system has demonstrated a high potential for quantifying the percentage of each grape variety.

Bulbarello et al. developed a hybrid electronic tongue including optical and electrochemical sensors able to evaluate bitterness in beverages fortified with plant extracts of green tea [89]. Two electrochemical sensors and one optical sensor showing independent and complementary signals towards epigallocatechin gallate and glucose, two of the most representative compounds found in fortified beverages and responsible for their final taste, were selected. Applying low- and mid-level data-fusion approaches, a preliminary PCA model and PLS regression models have been developed to provide two indices able to express the “bitterness” and “sweetness” intensity, the results being consistent with the declared composition of the soft drinks on the label.

Orlandi et al. applied different data-fusion strategies to merge the information brought by EE and ET sensing systems for the evaluation of grape ripening [90]. An amperometric ET was demonstrated to be sensitive to the concentration of the electroactive compounds of grape must [91], while an EE consisting of a common flatbed scanner was able to describe the color features of the must samples, which in turn are related to the concentration of the colored chemical species [92]. Thus, thanks to the synergy of the ET and EE responses, the application of data-fusion techniques (low level, mid-level with selected features, and mid-level with extracted features) allowed the Authors to consider the information brought by both the systems, improving the calibration models for a fast and easy determination of a significant number of parameters related to grape phenolic ripening.

Finally, Di Rosa et al. applied mid-level fusion methods, combining data from a potentiometric ET and a computer vision system, to classify different Sicilian honey varieties, achieving a satisfying recognizing percentage [93].

### 4.4. ET + EN + EE

Some applications of artificial sensors also imply the use of ET, EN, and EE altogether to acquire information from the samples; the resulting data are then jointly analyzed through data-fusion techniques (Table 5). In this manner, it is possible to gain a more comprehensive evaluation of the analyzed food matrix, somehow simulating human sensory perception. Indeed, the simultaneous use of EE, EN, and ET is often referred to as “electronic panel”, as this approach is able to mimic the human panel responses for sensory evaluation of the products [1,94].

Electronic panels composed of ET, EN, and EE were used to characterize organoleptic properties of extra virgin olive oils [28] and predict human sensory attributes of rice wine [29]. Both studies aimed at developing a combined sensor system able to replace, at least partially, human panel test of food products in order to speed up quality control of sensory properties. In Apetrei et al., the electronic panel was composed of an EN constructed using 13 MOS sensors, an ET based on modified carbon paste electrodes (CPE) sensors, and a spectrophotometer working in the 380–780 nm range acting as an EE [28]. Data obtained from the three devices were fused at the low level and used to develop classification and regression models to characterize extra virgin olive oil properties.

A similar approach was carried out by Ouyang et al. to predict sensory attributes of Chinese rice wines given by a panel test [29]. Trained experts attributed a score to color, taste, and aroma sensory properties to 75 samples of rice wine. The same samples were also analyzed using a portable EN system based on 10 MOS sensors, a commercial ET with seven different liquid cross-selective sensors, and a colorimeter. Data from the three sensors were combined together and used to develop multivariate calibration models able to predict the scores of the sensory characteristics of analyzed rice wine samples.

Xu et al. demonstrated that the combined use of EE, ET, and EN, together with proper data analysis approaches, generally outperforms single-sensor analysis in qualitative and quantitative evaluation of Longjing tea quality [95]. Longjing tea samples of different-quality grades were analyzed by means of reference wet chemistry methods in order to determine amino acids, catechins, polyphenols, and caffeine. Then, EN, ET, and EE signals of the tea samples were analyzed both separately and jointly in order to classify the samples according to quality grades and predict the content of the considered chemical components. Classification and regression models built with fused signals always outperformed those calculated considering the single sensors independently.

Prieto et al. highlighted an important advantage of using a combined electronic panel: EE, ET, and EN sensors are able to account for different aspects of the analyzed samples, and this information can be jointly used for a comprehensive characterization of the system under investigation [96]. In their study, the electronic panel was used to evaluate organoleptic properties of red wines prepared using different extraction techniques and micro-oxygenation methods and bottled using closures of different oxygen transmission rates. The results showed that EE and EN signals are able to describe the variability due to the oxygen transmission rate of the closure, while ET is more sensitive to the organoleptic properties related to polyphenol content and oxidation induced by micro-oxygenation. The combined use of the three sensing devices significantly improved the discrimination ability of the system to classify the samples according to the different vinification conditions.

The signals obtained from the three e-sensors are generally analyzed considering a low-level data-fusion approach, but some applications also involve mid-level data fusion. Buratti et al. used a mid-level data-fusion approach to combine EN, ET, and EE data in order to characterize edible olive oils, assessing their freshness in accelerated shelf-life tests [30]. PCA was used to extract relevant features of the three data blocks, and the resulting scores were merged together to obtain the e-senses fused-data matrix. This matrix was then analyzed by means of PCA to gain a comprehensive characterization of the analyzed oil samples based on the combined use of EE, ET, and EN. To improve model interpretability, the loading obtained from the PCA model of mid-level fused data were transformed back to the domain of original variables. In addition, mid-level fused data was also used to successfully classify the samples according to freshness.

## 5. Conclusions

Artificial sensing systems such as ENs, ETs, and EEs are gaining increasing relevance as analytical tools in the food industry for food quality assessment. Indeed, artificial sensors can provide information related to both sensory and chemical properties of the analyzed food matrix, allowing to minimize the need of sensory evaluations performed by panels of trained human experts or chemical determinations using sophisticated analytical devices. Furthermore, compared to traditional methods, artificial sensors have many advantages, including the possibility of analyzing a large number of samples in a short time and with limited reagent amounts, resulting in relevant economic savings.

The artificial sensors considered in this review account for different aspects of the analyzed matrix: ENs measure the presence and quantity of volatile compounds, ETs determine the concentration of chemical markers of interest in liquid samples, and EEs objectively evaluate color-related properties. All these aspects contribute to determine the quality of food products, and they are usually interconnected to each other.

Given these considerations, the possibility of coupling the signals obtained from different artificial sensors through data-fusion techniques has the great advantage of providing information about the different aspects of interest of the analyzed matrix, which in turn allows a more comprehensive characterization of the samples. Indeed, all the studies report a relevant increase in model performances when the signals resulting from the considered devices are jointly analyzed using data fusion compared to the results obtained with single artificial sensors.

Data fusion can be performed at different levels, i.e., low level, mid-level, and high level, and it is necessary to identify the proper data-fusion strategy for the problem at hand. The majority of the applications involve low-level and mid-level approaches, while the use of high-level data fusion is still very limited; indeed, only one application resulted from our survey. Generally, low-level data fusion is preferred as it is the easiest strategy, requiring minimal manipulation of each single block and a more straightforward interpretation of the results. On the other hand, mid-level data fusion usually performs better than low-level data fusion when the signals collected from the different devices contain many variables, because it allows one to retain the useful information of each data block in few descriptors, removing the noise at the same time.

The great advantages of fusing data collected from ENs, ETs, and EEs have also paved the way to the development of combined devices consisting of a single analytical instrument equipped with different types of artificial sensors whose signals are combined to provide one or more outputs of interest.

## Figures and Tables

**Table 1:** Biological senses involved in food quality assessment and analytical instruments suitable to represent the corresponding artificial senses.

Artificial Senses	Biological Senses	Sensory Properties	Analytical Instruments
Electronic tongue	Tongue	Taste/Flavor	Electrochemical sensors, optical sensors, gravimetric sensors
Electronic nose	Nose	Odor/Aroma	Electrochemical sensors, optical sensors, gravimetric sensors
Electronic eye	Eye	Color	Colorimeter, spectrophotometer, RGB camera

**Table 2:** List of publications related to the application of combined ET and EN.

Food Matrix	Aim of the Study	ET	EN	Data-Fusion Method	Ref.
Black tea	Quality assessment of black tea	5 electrodes made of 5 different noble metals	5 commercial MOS sensors	Mid-level of extracted features (wavelet)	[22]
Virgin olive oils	Characterize virgin olive oils from different geographical areas	4 electrodes of different metals	5 commercial MOS sensors	Low-level	[23]
Rice wines	Evaluating the marked ages of rice wines	3 types of modified electrodes with conducting polymer	12 MOS sensors	Low-level	[72]
Chinese Robusta coffees	Characterizationand classification of Chinese Robusta coffee cultivars	Commercial e-tongue	Commercial e-nose	Low-level	[73]
Black tea	Classification of different grade of black tea	5 electrodes made of 5 different noble metals	5 commercial MOS sensors	Mid-level of extracted features (wavelets)	[74]
*Orthosiphon stamineus*	Classification of *Orthosiphon stamineus*	7 commercial ion-selective sensors	Commercial e-nose	Low-level	[75]
Meat	Recognition of organoleptic characteristics of minced mutton adulterated with pork	Commercial taste system	Commercial e-nose	Low-level fusion and mid-level fusion	[76]
Cherry tomato juices	Authentication of fresh cherry tomato juices adulterated with overripe tomato juices	Commercial e-tongue	Commercial e-nose	Low-level; mid-level with selected features (PCA scores, F selection, stepwise selection)	[77]
Edible oil	Detection of the blending ratio of old frying oil and new edible-oil	Gold electrode	8 commercial gas sensors	Low-level	[78]
Mushroom	Detection of submerged fermentation	Commercial e-tongue	10 commercial MOS sensors	Low-level	[79]

**Table 3:** List of publications related to the application of combined EN and EE.

Food Matrix	Aim of the Study	EN	EE	Data-Fusion Method	Ref.
Pork meat	Determination of total volatile basic nitrogen content for evaluating pork freshness	11 commercial MOS sensors	CCD camera	Mid-level with selected features	[80]
Tilapia fillets	Characterization of fresh and spoiled tilapia fillets	12 commercial MOS sensors	CCD camera	Low-level	[81]
Longjing tea	Quality grading of tea samples	Commercial e-nose	CMOS camera	Mid-level with both feature extraction and feature selection High-level data fusion	[82]
Tomatoes	Prediction of ripening stage and quality parameters	10 MOS sensors	CCD camera	Mid-level, fusion of first PCs of each block	[83]
Strawberries	Evaluation of fungal contamination on strawberries during decay and determination of quality attributes	Commercial e-nose	Vis-NIR hyperspectral imaging system (400–1000 nm)	Low-level Mid-level with extracted features (PCA scores)	[84]
Pork meat	Quantification of intramuscular fat and peroxide value	Commercial e-nose	Vis-NIR hyperspectral imaging system (400–1000 nm)	Mid-level with extracted features (PCA scores after variable selection)	[85]

**Table 4:** List of publications related to the application of combined ET and EE.

Food Matrix	Aim of the Study	ET	EE	Data-Fusion Method	Ref.
Wine	Determination of quality parameters in red and white wines	Set of ISFET sensors	Spectrometer (200–1100 nm)	Mid-level with selected features	[86]
Wine	Characterization and quantification of grape varieties in red wines	Set of ISFET sensors	Spectrometer (200–1100 nm)	Mid-level with selected features	[87]
White grape juices	Discrimination of juices obtained from different grape varieties	Set of IFSET sensors	Lab-on-a-chip spectrophotometer (200–1100 nm)	Mid-level with selected features	[88]
Soft drinks fortified with extracts of green tea	Characterization of different formulations and prediction of sweetness and bitterness	2 screen printed sensors	UV–Vis spectrometer	Low-level and mid-level	[89]
Grape must	Quantification of the chemical parameters used to assess phenolic ripening in grapes	PEDOT electrode and SNGC-electrode	Flatbed scanner	Low-level; mid-level with selected features Mid-level with extracted features (PLS scores)	[90]

**Table 5:** List of publications related to the application of combined ET, EN, and EE.

Food Matrix	Aim of the Study	ET	EN	EE	Data-Fusion Method	Ref.
Extra virgin olive oils	Characterization of virgin olive oils from different varieties of olives and different degree of bitterness	Carbon paste Electrodes modified with olive oils	13 commercial MOS sensors	Spectrophotometer (380–780 nm)	Low-level	[28]
Rice wines	Prediction of human sensory attributes of rice wine	Commercial e-tongue	Portable e-nose	Colorimeter	Low-level	[29]
Olive oils	Characterization of edible olive oils and quality decay assessment of extra virgin olive oil and olive oil during shelf-life tests	Commercial e-tongue	Commercial e-nose	Spectrophotometer (380–780 nm)	Mid-level with extracted features (PCA scores)	[30]
Longjing green tea	Classification of quality grades and quantification of quality indices	Commercial e-tongue	Commercial e-nose	Colorimeter	Low-level	[95]
Wine	Discrimination of wines with different oxygen levels and antioxidant capabilities	Modified carbon paste electrodes	15 MOS sensors	UV–Vis spectrophotometer	Low-level	[96]

## Data Availability

Not applicable.
